# Supplementary material for: Deep neural networks explain spiking activity in auditory cortex
Source: PLoS Comput Biol. 2025 Aug 25;21(8):e1013334. doi: 10.1371/journal.pcbi.1013334 (PMC12404638; doi:10.1371/journal.pcbi.1013334)
Supplement: S1 Text — Noise correction of correlations (PDF) [file pcbi.1013334.s001.pdf]

S1 Text: Noise correction of correlations

Since neural responses are noisy (no two presentations of a stimulus elicit the same response), even an optimal encoding model cannot completely explain the neural response. It is therefore customary, when reporting the performance of an encoding model, to normalize the correlations of the model with neural activity ("model-neuron correlations") by the correlations achievable by an *optimal* model. The latter cannot be computed directly; however, they can be estimated via the *trial-to-trial* neural correlations, i.e. the correlations between different responses to the same stimulus. Intuitively, trial-to-trial correlations involve two noisy variables, whereas model-neuron correlations involve only one (the model is deterministic); and so the latter is just the square root of the former. The result is well known but for completeness we prove it here precisely.

Estimating model-neuron correlations via trial-to-trial neural correlations. Assume that neural responses to the same stimulus on different trials are independent and identically distributed (i.i.d.), conditioned on that stimulus (see the probabilistic graphical model at right). We call the (unobserved) expected value X:

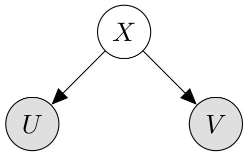

$$\mathbb{E}_{U|X}[U|X] = \mathbb{E}_{V|X}[V|X] =: X \tag{1}$$

for the responses, U and V, on two different trials. Although U and V are conditionally uncorrelated by hypothesis, marginalizing out X induces correlation between them. To work out this correlation we need their covariance, which by the law of total covariance is

$$\begin{aligned}\mathrm{Cov}_{U,V}[U,V] &= \mathbb{E}_{U,V}\left[\mathrm{Cov}_{U,V|X}[U,V|X]\right] + \mathrm{Cov}_X\left[\mathbb{E}_{U|X}[U|X], \mathbb{E}_{V|X}[V|X]\right] \\ &= 0 + \mathrm{Cov}_X[X,X] = \mathrm{Var}_X[X].\end{aligned} \tag{2}$$

The first term vanishes because we have assumed that trials are conditionally independent; and the second term simplifies according to Eq. 1. Intuitively, fluctuations that are shared between U and V are due entirely to fluctuations in X, their common mean.
Now we turn to the model-neuron correlation. We are interested in the ceiling of performance, so we consider the optimal model, which will recover exactly the noise-free mean response to the stimulus, X. To compute its correlation with a neural response U, we begin again with the covariance, which by the law of total covariance is

$$\begin{aligned}\mathrm{Cov}_{X,U}[X,U] &= \mathbb{E}_X\left[\mathrm{Cov}_{X,U|X}[X,U|X]\right] + \mathrm{Cov}_X\left[\mathbb{E}_{U|X}[U|X], \mathbb{E}_{X|X}[X|X]\right] \\ &= 0 + \mathrm{Cov}_X[X,X] = \mathrm{Var}_X[X].\end{aligned} \tag{3}$$

This time the first term vanishes because U has nothing left to covary with once X is fixed.
Comparing Eqs. 2 and 3, we see that the trial-to-trial covariance and the (optimal) model-neuron covariance are identical. However, the correlation coefficient reports this shared fluctuation as a fraction of the total fluctuations of the two variables. Hence the trial-to-trial correlation is

$$\mathrm{Corr}[U,V] = \frac{\mathrm{Cov}_{U,V}[U,V]}{\sqrt{\mathrm{Var}_U[U]\mathrm{Var}_V[V]}} = \frac{\mathrm{Var}_X[X]}{\mathrm{Var}_U[U]} = \frac{\sigma_x^2}{\sigma_u^2}. \tag{4}$$

The second equality follows because the noise is assumed identically distributed. Notice that this quantity is necessarily less than one because the variance of U includes all the variation of X, plus the variation due to noise. In contrast, the model-neuron correlation is

$$\mathrm{Corr}[X,U] = \frac{\mathrm{Cov}_{X,U}[X,U]}{\sqrt{\mathrm{Var}_X[X]\mathrm{Var}_U[U]}} = \frac{\mathrm{Var}_X[X]}{\sigma_x\sigma_u} = \frac{\sigma_x}{\sigma_u}. \tag{5}$$

This quantity is likewise less than 1.
Since the maximum possible model-neuron correlation (Eq. 5) is less than 1, it is sensible to report *empirical* model-neuron correlations as a fraction of the maximum model-neuron correlation (scaling them back up to a maximum of 1). As noted at the outset, it is impossible to compute the latter, since we don't have access to the optimal model. But comparing Eqs. 5 and 4, we see that $\mathrm{Corr}[X,U] = \sqrt{\mathrm{Corr}[U,V]}$ (as expected). So we could use the square root of the trial-to-trial correlation in place of the optimal (maximum) model-neuron correlation.

Estimating the true trial-to-trial correlation with sample correlations. In fact, the *true* trial-to-trial correlation is also unavailable, so in practice we must estimate it with a *sample* correlation. In particular, we assume that the binned spike counts (U_n, $n = 1, \ldots, N$) from a single pass through all stimuli are i.i.d., and likewise for some other pass (V_n) through the same stimuli. If both passes through the stimuli are in the same order (so that X is the same on both passes), we can estimate the (marginal) correlation with the sample correlation between U_n and V_n. For any finite N, the sample correlation will only approximate the true correlation; therefore we use a resampling procedure across several complete passes through the stimuli (see **Methods**) to generate a distribution of sample correlations. Furthermore, the binned spikes and the responses across trials may not be perfectly i.i.d. in practice, due to slow fluctuations in attention, background activity, and the like. This will in general broaden the distribution. We let our normalizer be the median of this distribution (rather than mean, which is more sensitive to outliers).
