# Supplementary material for: Deep neural networks explain spiking activity in auditory cortex
Source: PLoS Comput Biol. 2025 Aug 25;21(8):e1013334. doi: 10.1371/journal.pcbi.1013334 (PMC12404638; doi:10.1371/journal.pcbi.1013334)
Supplement: S2 Text — Pretrained networks (PDF) [file pcbi.1013334.s002.pdf]

S2 Text: Pretrained networks

To investigate neural responses at fine temporal resolutions, we selected a diverse set of pretrained neural networks that span various architectures and training paradigms. A key criterion in our model selection was the availability of pretrained weights, allowing us to focus on analyzing their representations rather than training them from scratch. S1 Table summarizes the models included in our study. Among these, WAV2LETTER is the only model we trained from scratch; all others are open-source and publicly available.[a]

Training Details for WAV2LETTER

WAV2LETTER consists of 15 convolutional layers, each followed by batch normalization, ReLU non-linearity, and dropout. Each layer contains 250 feature maps. The model was trained on the full 960-hour LibriSpeech dataset using the Adam optimizer with a learning rate of 0.001 and a batch size of 8.

Performance of Pretrained ANNs

The pretrained networks analyzed in this study were originally trained on different datasets, each with varying amounts and types of data. To enable a fair comparison and to assess their generalization capabilities, we evaluated all models on three benchmark datasets that are distinct from their respective training sets. This evaluation provides insight into how well these models generalize beyond their original training domains. The word error rates (WERs) for these evaluations are reported in S6 Table.

---

[a] For the training code and weights for WAV2LETTER, see the **Data availability statement**; for all others, see the references in the main text.
