# Supplementary material for: Deep neural networks explain spiking activity in auditory cortex
Source: PLoS Comput Biol. 2025 Aug 25;21(8):e1013334. doi: 10.1371/journal.pcbi.1013334 (PMC12404638; doi:10.1371/journal.pcbi.1013334)
Supplement: S1 Table — ANN training details. (PDF) [file pcbi.1013334.s003.pdf]

S1 Table. ANN training details.

model	input	training data	training type
WAV2LETTER	raw waveform	LibriSpeech (960 Hrs.)	supervised
WAV2VEC2	raw waveform	LibriSpeech (960 Hrs.)	self-supervised
SPEECH2TEXT	spectrogram	LibriSpeech (960 Hrs.)	weakly-supervised
WHISPER(tiny)	spectrogram	multi-lingual audio (650,000 Hrs.)	weakly-supervised
WHISPER(base)	spectrogram	same as WHISPER(tiny)	weakly-supervised
DEEPSPEECH2	spectrogram	LibriSpeech, WSJ, Switchboard (12,000 Hrs.)	supervised
