# Supplementary material for: Deep neural networks explain spiking activity in auditory cortex
Source: PLoS Comput Biol. 2025 Aug 25;21(8):e1013334. doi: 10.1371/journal.pcbi.1013334 (PMC12404638; doi:10.1371/journal.pcbi.1013334)
Supplement: S2 Table — Recording probes used in experiments. (PDF) [file pcbi.1013334.s004.pdf]

S2 Table. Recording probes used in experiments.

type	# channels	layout
Tungsten electrode	1	single-channel
ATLAS Neuro E-16-S1	16	linear array
NeuroNexus A1x32-Poly3	32	Poly3
NeuroNexus A1x48-Poly2	48	Poly2
Cambridge ASSY-77 H2	64	2-shank linear
NeuroNexus A4x4-tet	64	4-shank tetrode
