# Supplementary material for: Deep neural networks explain spiking activity in auditory cortex
Source: PLoS Comput Biol. 2025 Aug 25;21(8):e1013334. doi: 10.1371/journal.pcbi.1013334 (PMC12404638; doi:10.1371/journal.pcbi.1013334)
Supplement: S1 Fig — Noise correction and unit selection. A: Procedure for computing the true and null distributions of trial-to-trial correlations. Both distributions are computed using a set of stimuli with multiple presentations. For each stimulus in the set, a distinct pair of trials is randomly selected (STEP 1) and the responses concatenated in random order to form two response sequences, U and V (STEP 2). For computing the null distribution only, V is circularly shifted by half its length (STEP 3), which preserves the marginal response statistics for each channel but destroys any possible temporal correlations with U. Finally, the correlation coefficient between U and V is computed (STEP 4). This process is repeated 100,000 times to obtain distributions (true and null) of correlation coefficients. B: Number of multi-units with at least Î´ standard deviations between their true and null distributions, as a function of delta. Results are nearly identical if the inter-mean (blue) or inter-median (orange) distance is used. C: True (blue) and null (gray) distributions of correlations for three randomly selected channels whose means (indicated by dotted vertical lines) are separated by a gap of δ=0.1, 0.5 and 1.0 times the standard deviation of the null distribution. For all three multi-units, the true distribution is shifted significantly to the right of the null distribution under a Wilcoxon rank-sum test (p < 0.05), despite overlapping very heavily (see esp. δ=0.1). (PDF) [file pcbi.1013334.s005.pdf]

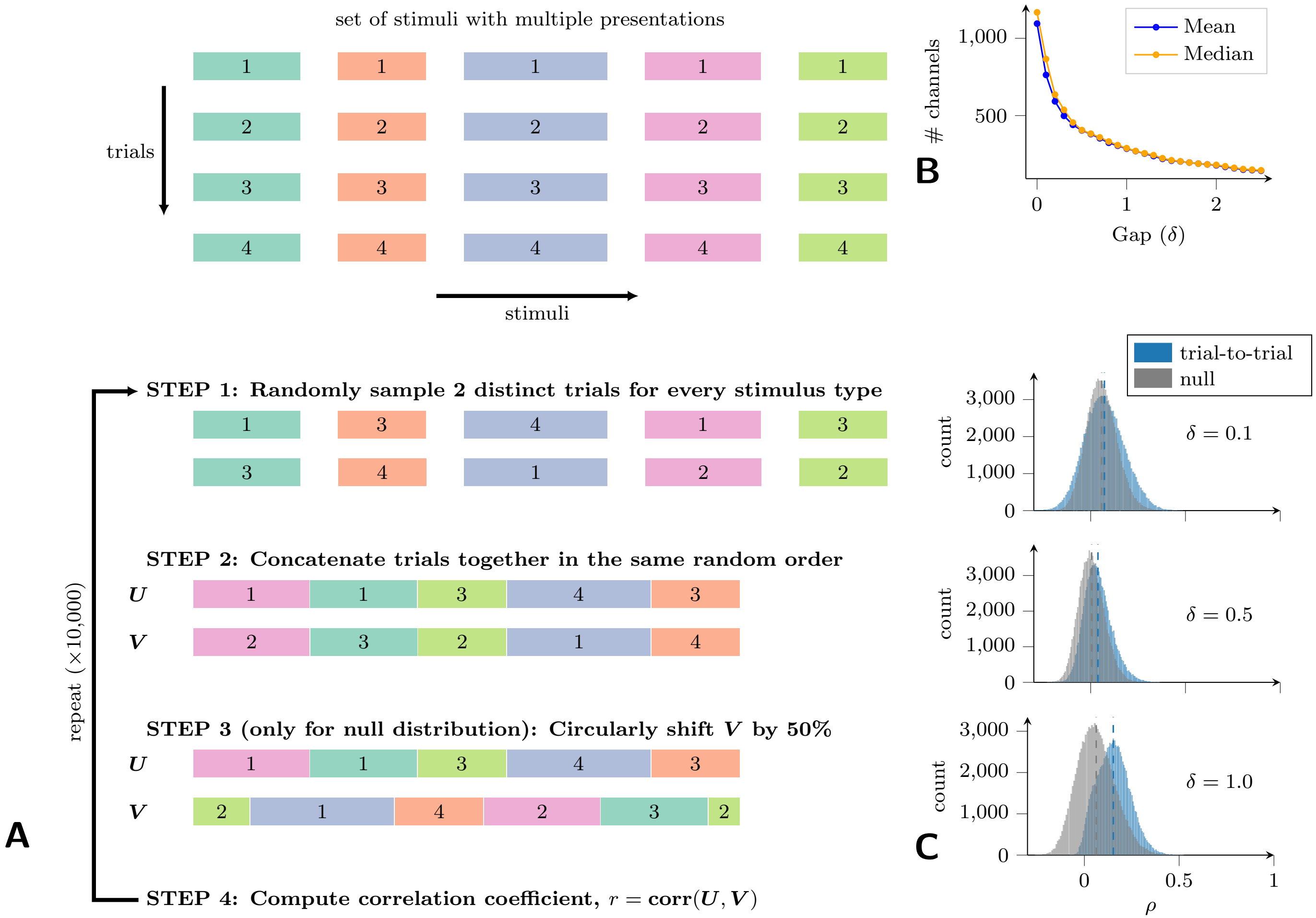

S1 Fig. Noise correction and unit selection. A: Procedure for computing the true and null distributions of trial-to-trial correlations. Both distributions are computed using a set of stimuli with multiple presentations. For each stimulus in the set, a distinct pair of trials is randomly selected (STEP 1) and the responses concatenated in random order to form two response sequences, $\boldsymbol{U}$ and $\boldsymbol{V}$ (STEP 2). For computing the null distribution only, $\boldsymbol{V}$ is circularly shifted by half its length (STEP 3), which preserves the marginal response statistics for each channel but destroys any possible temporal correlations with $\boldsymbol{U}$. Finally, the correlation coefficient between $\boldsymbol{U}$ and $\boldsymbol{V}$ is computed (STEP 4). This process is repeated 100,000 times to obtain distributions (true and null) of correlation coefficients. B: Number of multi-units with at least δ standard deviations between their true and null distributions, as a function of delta. Results are nearly identical if the inter-mean (blue) or inter-median (orange) distance is used. C: True (blue) and null (gray) distributions of correlations for three randomly selected channels whose means (indicated by dotted vertical lines) are separated by a gap of $\delta = 0.1$, 0.5 and 1.0 times the standard deviation of the null distribution. For all three multi-units, the true distribution is shifted significantly to the right of the null distribution under a Wilcoxon rank-sum test ($p < 0.05$), despite overlapping very heavily (see esp. $\delta = 0.1$).
