# Supplementary material for: Deep neural networks explain spiking activity in auditory cortex
Source: PLoS Comput Biol. 2025 Aug 25;21(8):e1013334. doi: 10.1371/journal.pcbi.1013334 (PMC12404638; doi:10.1371/journal.pcbi.1013334)
Supplement: S3 Table — Number of tuned neurons. See Materials and methods for criteria for being tuned and well-tuned. Stimulus classes are TIMIT sentences (“speech”) and monkey vocalizations (“mVox”). (PDF) [file pcbi.1013334.s006.pdf]

S3 Table. Number of tuned neurons. See **Materials and methods** for criteria for being tuned and well-tuned. Stimulus classes are TIMIT sentences ("speech") and monkey vocalizations ("mVox").

	**tuned**			**well-tuned**		
**stimulus class**	speech	mVox	both	speech	mVox	both
**core**	707	701	580	300	315	246
**non-primary**	488	530	341	104	174	78
**all**	1195	1231	921	404	489	324
