# Supplementary material for: Deep neural networks explain spiking activity in auditory cortex
Source: PLoS Comput Biol. 2025 Aug 25;21(8):e1013334. doi: 10.1371/journal.pcbi.1013334 (PMC12404638; doi:10.1371/journal.pcbi.1013334)
Supplement: S4 Table — ANN layer properties. RF: receptive field; Ts: sampling period. (PDF) [file pcbi.1013334.s007.pdf]

S4 Table. ANN layer properties. RF: receptive field; T_s: sampling period.

model	layer	type	RF (ms)	T_s (ms)	# units
wav2letter (modified)	0	conv	1.94	1.25	250
	1	conv	4.44	2.50	250
	2	conv	9.44	5.00	250
	3	conv	19.4	10.0	250
	4	conv	39.4	20.0	250
	5	conv	79.4	20.0	250
	6	conv	119.4	20.0	250
	7	conv	159.4	20.0	250
	8	conv	279.4	20.0	250
	9	conv	399.4	20.0	250
	10	conv	519.4	20.0	250
	11	conv	639.4	20.0	250
	12	conv	1239.4	20.0	2000
	13	conv	1239.4	20.0	2000
wav2vec2	0	conv	0.625	0.312	512
	1	conv	1.25	0.625	512
	2	conv	2.50	1.25	512
	3	conv	5.00	2.50	512
	4	conv	10.0	5.00	512
	5	conv	15.0	10.0	512
	6	conv	25.0	20.0	512
	7–20	attention	2565.0	20.0	768
speech2text	0	conv	65.0	20.0	1024
	1	conv	145.0	40.0	2048
	2–13	attention	full	40.0	1024
Whisper (tiny)	0	conv	45.0	10.0	384
	1	conv	65.0	20.0	384
	2–5	attention	full	20.0	384
Whisper (base)	0	conv	45.0	10.0	512
	1	conv	65.0	20.0	512
	2–7	attention	full	20.0	512
DeepSpeech2	0	conv	120.0	20.0	2592
	1	conv	320.0	20.0	1312
	2–6	recurrent	full	20.0	2048
