# Supplementary material for: Deep neural networks explain spiking activity in auditory cortex
Source: PLoS Comput Biol. 2025 Aug 25;21(8):e1013334. doi: 10.1371/journal.pcbi.1013334 (PMC12404638; doi:10.1371/journal.pcbi.1013334)
Supplement: S5 Table — Comparison of different spectrogram computations. (PDF) [file pcbi.1013334.s008.pdf]

S5 Table. Comparison of different spectrogram computations.

	**DeepSpeech2**	**Speech2Text**	**Whisper**	**Librosa**
**freq. scale**	linear	mel	mel	mel
**ampl. scale**	log	log	log	linear
**mel Scale**	none	Kaldi	Slaney	Slaney
**freq. range (Hz)**	0–8000	20–8000	0–8000	0–8000
**window**	Hamming	Povey	Hann	Hann
