# Supplementary material for: Deep neural networks explain spiking activity in auditory cortex
Source: PLoS Comput Biol. 2025 Aug 25;21(8):e1013334. doi: 10.1371/journal.pcbi.1013334 (PMC12404638; doi:10.1371/journal.pcbi.1013334)
Supplement: S2 Fig — Comparison of STRF-neuron correlations for various choices of spectrogram computation. Each box depicts the distribution of correlations across multi-units for a particular choice of spectrogram. The median, middle quartiles, and 5th/95th percentiles are indicated with a black line, shading, and whiskers (resp.). Results are shown separately for each stimulus type, speech and monkey-vocalization (vox). (PDF) [file pcbi.1013334.s009.pdf]

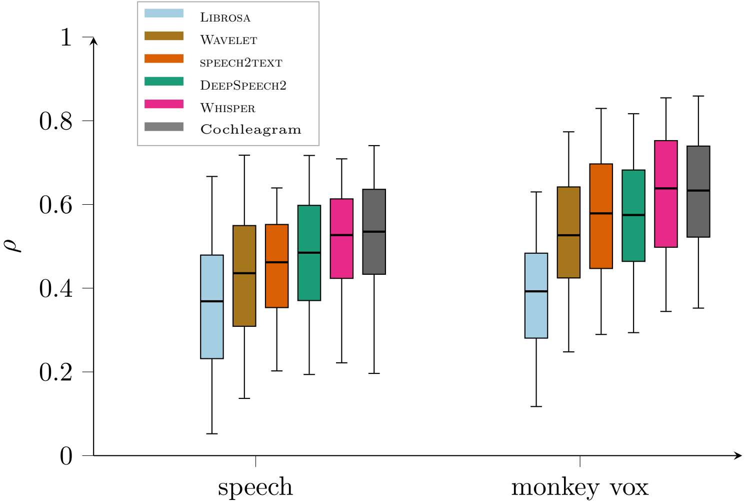

S2 Fig. Comparison of STRF-neuron correlations for various choices of spectrogram computation. Each box depicts the distribution of correlations across multi-units for a particular choice of spectrogram. The median, middle quartiles, and 5th/95th percentiles are indicated with a black line, shading, and whiskers (resp.). Results are shown separately for each stimulus type, speech and monkey-vocalization (vox).
