# Supplementary material for: Deep neural networks explain spiking activity in auditory cortex
Source: PLoS Comput Biol. 2025 Aug 25;21(8):e1013334. doi: 10.1371/journal.pcbi.1013334 (PMC12404638; doi:10.1371/journal.pcbi.1013334)
Supplement: S6 Table — ANN task performance. Comparison of pretrained models on speech recognition tasks, reported as word error rates (WER, %) across three test datasets. All test sets are distinct from the training data used for each model to ensure a fair comparison. (PDF) [file pcbi.1013334.s010.pdf]

S6 Table. ANN task performance. Comparison of pretrained models on speech recognition tasks, reported as word error rates (WER, %) across three test datasets. All test sets are distinct from the training data used for each model to ensure a fair comparison.

**model**	**WER (%)**		
	**TED-LIUM 3**	**Common Voice 5.1**	**VoxPopuli**
wav2letter	59.98	86.23	81.42
wav2vec2	14.07	36.20	21.58
speech2text	15.51	36.58	21.18
Whisper (tiny)	7.81	31.58	12.90
Whisper (base)	**5.95**	**22.36**	**10.36**
DeepSpeech2	35.59	64.10	49.67
