# Supplementary material for: Deep neural networks explain spiking activity in auditory cortex
Source: PLoS Comput Biol. 2025 Aug 25;21(8):e1013334. doi: 10.1371/journal.pcbi.1013334 (PMC12404638; doi:10.1371/journal.pcbi.1013334)
Supplement: S3 Fig — Model-neuron correlation ratios. All subpanels show the correlations between model predictions and the multi-unit activity they are supposed to predict, each normalized by the corresponding STRF-neuron correlation. A: Model-neuron correlation ratios for speech (TIMIT) stimuli. Bar plot: median (across multi-units) correlation ratio for the STRF (gray, necessarily 1.0) and of the best layers of each of the neural networks, both trained (dark colors) and untrained (light colors). Line plots: the distributions of correlation ratios as a function of ANN layer for each of six trained networks (colored) and their untrained counterparts (gray). The median (solid line) and interquartile range (shaded region) are shown. The layer type is indicated along the top of each plot: convolutional (brown), self-attention (blue), and recurrent (light blue). B: The same as A but using monkey vocalizations for stimuli. (PDF) [file pcbi.1013334.s011.pdf]

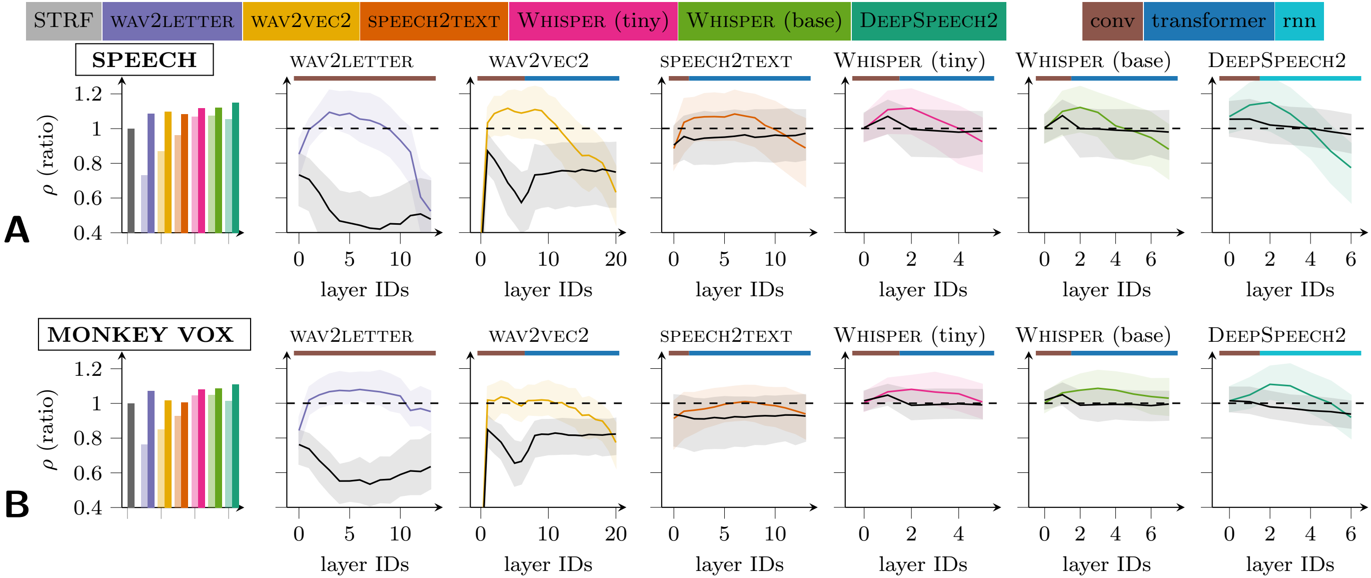

S3 Fig. Model-neuron correlation ratios. All subpanels show the correlations between model predictions and the multi-unit activity they are supposed to predict, each normalized by the corresponding STRF-neuron correlation. A: Model-neuron correlation ratios for speech (TIMIT) stimuli. Bar plot: median (across multi-units) correlation ratio for the STRF (gray, necessarily 1.0) and of the best layers of each of the neural networks, both trained (dark colors) and untrained (light colors). Line plots: the distributions of correlation ratios as a function of ANN layer for each of six trained networks (colored) and their untrained counterparts (gray). The median (solid line) and interquartile range (shaded region) are shown. The layer type is indicated along the top of each plot: convolutional (brown), self-attention (blue), and recurrent (light blue). B: The same as A but using monkey vocalizations for stimuli.
