# Supplementary material for: Deep neural networks explain spiking activity in auditory cortex
Source: PLoS Comput Biol. 2025 Aug 25;21(8):e1013334. doi: 10.1371/journal.pcbi.1013334 (PMC12404638; doi:10.1371/journal.pcbi.1013334)
Supplement: S4 Fig — Model-neuron correlations for two different trained versions of wav2vec2. All subpanels show correlations between model predictions and the multi-unit activity they are supposed to predict. A: Model-neuron correlations for speech (TIMIT) stimuli for wav2vec2, either trained as in the main text (yellow), or only pre-trained on AudioSet (red). B: The same as A but using monkey vocalizations for stimuli. (PDF) [file pcbi.1013334.s012.pdf]

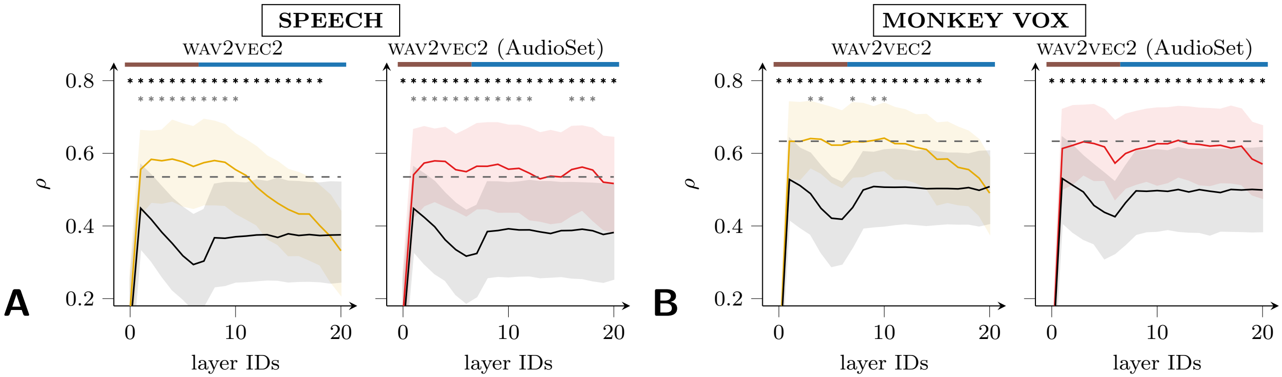

S4 Fig. Model-neuron correlations for two different trained versions of WAV2VEC2. All subpanels show correlations between model predictions and the multi-unit activity they are supposed to predict. A: Model-neuron correlations for speech (TIMIT) stimuli for WAV2VEC2, either trained as in the main text (yellow), or only pre-trained on AudioSet (red). B: The same as A but using monkey vocalizations for stimuli.
