# Supplementary material for: Deep neural networks explain spiking activity in auditory cortex
Source: PLoS Comput Biol. 2025 Aug 25;21(8):e1013334. doi: 10.1371/journal.pcbi.1013334 (PMC12404638; doi:10.1371/journal.pcbi.1013334)
Supplement: S5 Fig — Maximum (across layers) median ANN-neuron correlation vs. ANN word error rate on three ASR data sets. Each point corresponds to an ANN (color scheme as throughout). Circles: speech (TIMIT) stimuli; plus signs: monkey vocalizations. (PDF) [file pcbi.1013334.s013.pdf]

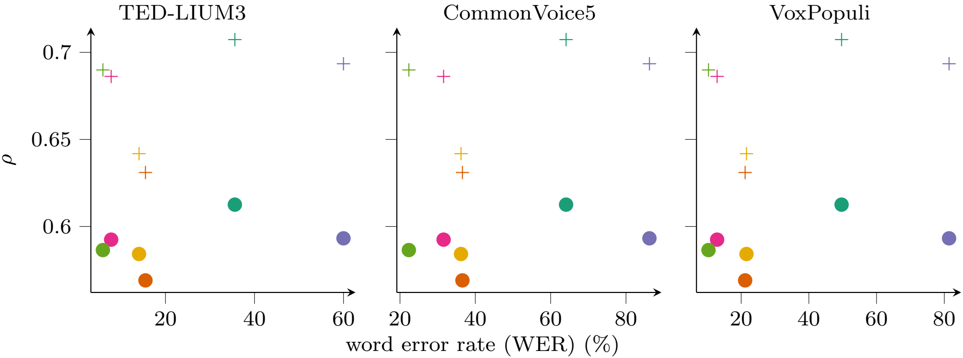

S5 Fig. Maximum (across layers) median ANN-neuron correlation vs. ANN word error rate on three ASR data sets. Each point corresponds to an ANN (color scheme as throughout). Circles: speech (TIMIT) stimuli; plus signs: monkey vocalizations.
