# Supplementary material for: Deep neural networks explain spiking activity in auditory cortex
Source: PLoS Comput Biol. 2025 Aug 25;21(8):e1013334. doi: 10.1371/journal.pcbi.1013334 (PMC12404638; doi:10.1371/journal.pcbi.1013334)
Supplement: S6 Fig — Distributions of most predictive layers (normalized) for primary (blue) and non-primary (orange) neurons. Histograms and corresponding kernel density estimates are shown as a function of network depth (from shallowest to deepest) for all neurons, separately for each ANN. A: TIMIT stimuli. B: Monkey-vocalization stimuli. For each pair of distributions, a Wilcoxon rank-sum test was used to determine if the non-primary neurons “prefer” deeper layers of the network (* for p<0.05, * * for p<0.01, * ** for p<0.001). (PDF) [file pcbi.1013334.s014.pdf]

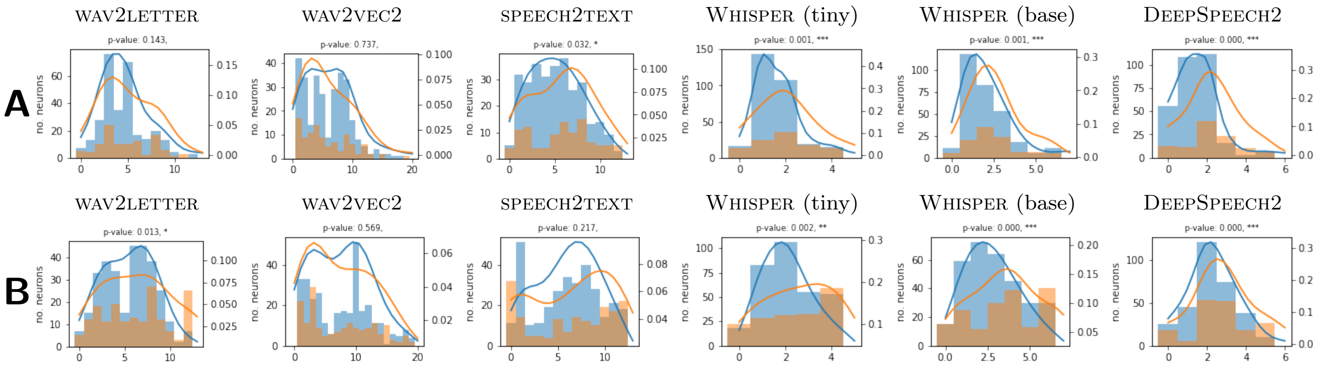

S6 Fig. Distributions of most predictive layers (normalized) for primary (blue) and non-primary (orange) neurons. Histograms and corresponding kernel density estimates are shown as a function of network depth (from shallowest to deepest) for all neurons, separately for each ANN. A: TIMIT stimuli. B: Monkey-vocalization stimuli. For each pair of distributions, a Wilcoxon rank-sum test was used to determine if the non-primary neurons "prefer" deeper layers of the network (* for $p < 0.05$, ** for $p < 0.01$, *** for $p < 0.001$).
