# Supplementary material for: Deep neural networks explain spiking activity in auditory cortex
Source: PLoS Comput Biol. 2025 Aug 25;21(8):e1013334. doi: 10.1371/journal.pcbi.1013334 (PMC12404638; doi:10.1371/journal.pcbi.1013334)
Supplement: S7 Fig — Model-neuron correlations for cochresnet50 with two different types of “untrained” model. All subpanels show correlations between model predictions and the multi-unit activity they are supposed to predict. A: Model-neuron correlations for speech (TIMIT) stimuli. The untrained networks (black line and shading) were created either by re-initializing the weights or by permuting the weights of the trained model. B: The same as A but using monkey vocalizations for stimuli. (PDF) [file pcbi.1013334.s015.pdf]

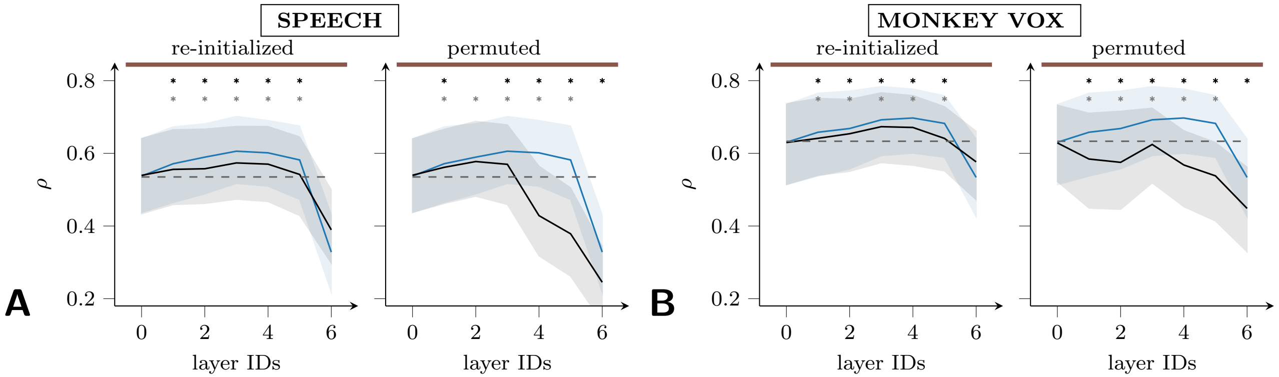

S7 Fig. Model-neuron correlations for COCHRESNET50 with two different types of "untrained" model. All subpanels show correlations between model predictions and the multi-unit activity they are supposed to predict. A: Model-neuron correlations for speech (TIMIT) stimuli. The untrained networks (black line and shading) were created either by re-initializing the weights or by permuting the weights of the trained model. B: The same as A but using monkey vocalizations for stimuli.
