# Supplementary material for: Deep neural networks explain spiking activity in auditory cortex
Source: PLoS Comput Biol. 2025 Aug 25;21(8):e1013334. doi: 10.1371/journal.pcbi.1013334 (PMC12404638; doi:10.1371/journal.pcbi.1013334)
Supplement: S8 Fig — Model-neuron correlations using δ=1.0 as the inclusion criterion. All subpanels show correlations between model predictions and the multi-unit activity they are supposed to predict. A: Model-neuron correlations for speech (TIMIT) stimuli. Bar plot: median (across multi-units) correlation of the STRF (gray) and of the best layers of each of the neural networks, both trained (dark colors) and untrained (light colors). Line plots: the distributions of correlations as a function of ANN layer for each of six trained networks (colored) and their untrained counterparts (gray). The median (solid line) and interquartile range (shaded region) are shown. At starred layers, the trained ANN is significantly superior to its untrained counterpart (top row of stars) or a STRF (bottom row of stars; Wilcoxon signed-rank test with p<0.01). To control the false discovery rate, we apply the Benjamini–Hochberg correction separately for each model. The layer type is indicated along the top of each plot: convolutional (brown), self-attention (blue), and recurrent (light blue). B: The same as A but using monkey vocalizations for stimuli. (PDF) [file pcbi.1013334.s016.pdf]

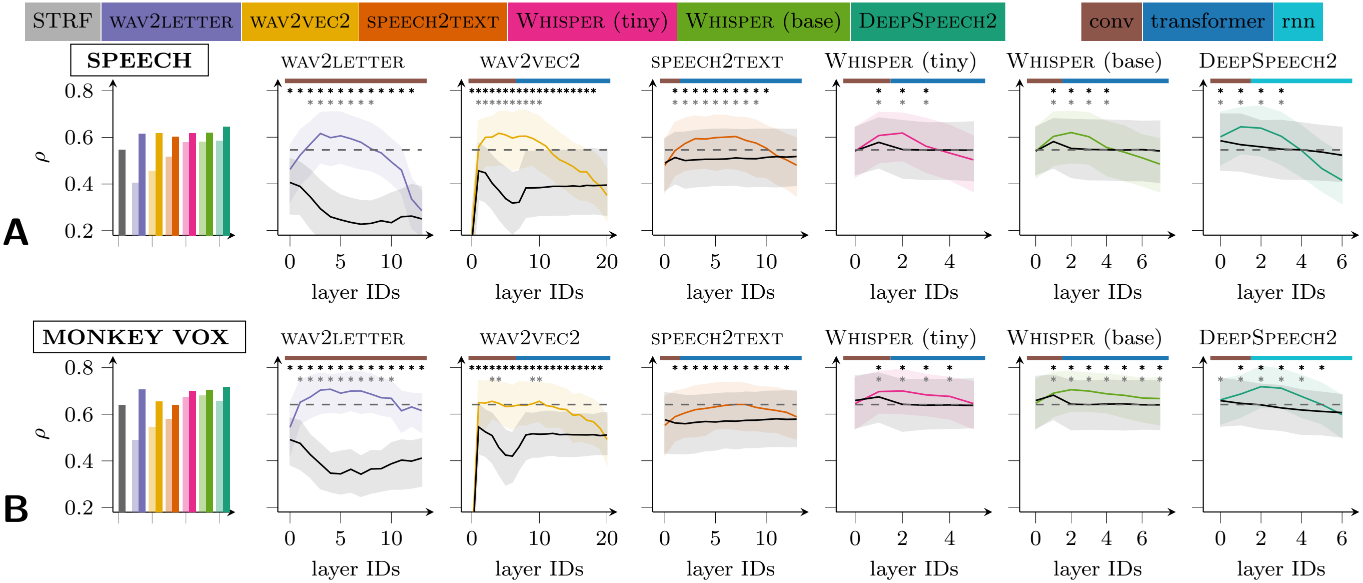

S8 Fig. Model-neuron correlations using δ=1.0 as the inclusion criterion. All subpanels show correlations between model predictions and the multi-unit activity they are supposed to predict. A: Model-neuron correlations for speech (TIMIT) stimuli. Bar plot: median (across multi-units) correlation of the STRF (gray) and of the best layers of each of the neural networks, both trained (dark colors) and untrained (light colors). Line plots: the distributions of correlations as a function of ANN layer for each of six trained networks (colored) and their untrained counterparts (gray). The median (solid line) and interquartile range (shaded region) are shown. At starred layers, the trained ANN is significantly superior to its untrained counterpart (top row of stars) or a STRF (bottom row of stars; Wilcoxon signed-rank test with $p < 0.01$). To control the false discovery rate, we apply the Benjamini–Hochberg correction separately for each model. The layer type is indicated along the top of each plot: convolutional (brown), self-attention (blue), and recurrent (light blue). B: The same as A but using monkey vocalizations for stimuli.
