# Supplementary material for: Deep neural networks explain spiking activity in auditory cortex
Source: PLoS Comput Biol. 2025 Aug 25;21(8):e1013334. doi: 10.1371/journal.pcbi.1013334 (PMC12404638; doi:10.1371/journal.pcbi.1013334)
Supplement: S9 Fig — Distributions of model-neuron correlations as a function of maximum frequency of the model predictions using Î´=1.0 as the inclusion criterion. Median (solid line) and interquartile range (shaded) are indicated. For each ANN, results are shown only for the layer that was most predictive at 50-ms bins. First, the layer’s responses were low-pass filtered at the frequency indicated on the horizontal axis. Then a linear readout (TRF) was fit to predict spiking activity binned at 20 ms. The vertical axis shows the resulting distribution of correlations on the test set. The red star indicates the cut-off frequency yielding the largest median (across multi-units) correlation; black dots indicate frequencies yielding correlation distributions indistinguishable from that at the red star (Wilcoxon signed-rank test with p < 0.01). (PDF) [file pcbi.1013334.s017.pdf]

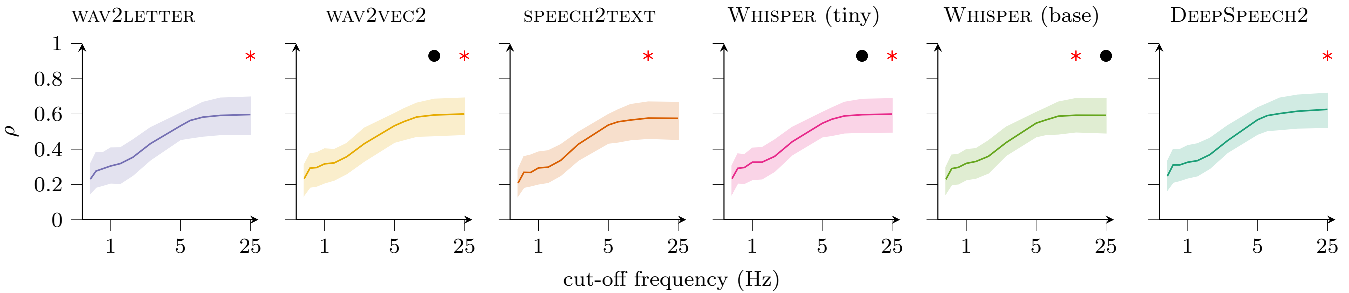

S9 Fig. Distributions of model-neuron correlations as a function of maximum frequency of the model predictions using δ=1.0 as the inclusion criterion. Median (solid line) and interquartile range (shaded) are indicated. For each ANN, results are shown only for the layer that was most predictive at 50-ms bins. First, the layer's responses were low-pass filtered at the frequency indicated on the horizontal axis. Then a linear readout (TRF) was fit to predict spiking activity binned at 20 ms. The vertical axis shows the resulting distribution of correlations on the test set. The red star indicates the cut-off frequency yielding the largest median (across multi-units) correlation; black dots indicate frequencies yielding correlation distributions indistinguishable from that at the red star (Wilcoxon signed-rank test with $p < 0.01$).
