# Supplementary material for: Deep neural networks explain spiking activity in auditory cortex
Source: PLoS Comput Biol. 2025 Aug 25;21(8):e1013334. doi: 10.1371/journal.pcbi.1013334 (PMC12404638; doi:10.1371/journal.pcbi.1013334)
Supplement: S10 Fig — Distributions of most predictive layers (normalized) for primary (blue) and non-primary (orange) multi-unit activity using δ=1.0 as the inclusion criterion. Histograms and corresponding kernel density estimates are shown as a function of network depth (from shallowest to deepest), pooled across all neurons and all six ANNs. The distribution of preferred layers is significantly “deeper” for non-primary than primary neurons (Wilcoxon rank-sum test, p < 0.001). (PDF) [file pcbi.1013334.s018.pdf]

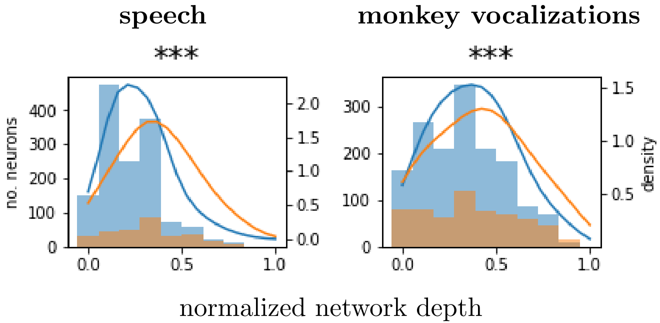

S10 Fig. Distributions of most predictive layers (normalized) for primary (blue) and non-primary (orange) multi-unit activity using δ=1.0 as the inclusion criterion. Histograms and corresponding kernel density estimates are shown as a function of network depth (from shallowest to deepest), pooled across all neurons and all six ANNs. The distribution of preferred layers is significantly "deeper" for non-primary than primary neurons (Wilcoxon rank-sum test, $p < 0.001$).
