# Supplementary material for: Deep neural networks explain spiking activity in auditory cortex
Source: PLoS Comput Biol. 2025 Aug 25;21(8):e1013334. doi: 10.1371/journal.pcbi.1013334 (PMC12404638; doi:10.1371/journal.pcbi.1013334)
Supplement: Attachment — Submitted filename: DNNEACreplyToReviewers.pdf [file pcbi.1013334.s019.pdf]

Deep Neural Networks Explain Spiking Activity in Auditory Cortex: Replies to reviewers

We thank all the reviewers for very close readings of our manuscript and their constructive feedback.

General replies

We made three significant changes to the manuscript that are of general interest and we cover those first.

Selecting tuned neurons. Multiple reviewers raised concerns about our process for selecting tuned neurons. Reviewer 1 felt that our justification for including only those neurons beyond the 90th percentile of the null distribution was arbitrary, and Reviewer 3 questioned the validity of the null distribution itself (in particular, the assumption of Poisson-distributed spike counts). We note that the use of arbitrary inclusion criteria is common in the literature (e.g., Kell and colleagues [1] include only the top 5% of speech-responsive voxels in their analyses), but concede that our manuscript could be made stronger on this front.

Accordingly, **we have adopted a new method for computing the null distribution, namely, computing trial-to-trial correlations between responses that have been circularly shifted by half the sequence length**. This retains the marginal statistics of neural responses while breaking correlation structure. Every neuron (or more properly, multi-unit) therefore has its own null distribution. The choice of how far out on this null distribution a multi-unit's mean or median trial-to-trial correlations must be in order to count as "tuned" is still somewhat arbitrary, but can be improved by plotting the number of included neurons as a function of this distance. Fig. R1B shows the result for all multi-units recorded during our experiments. We observe an elbow in the curve at about 0.5 standard deviations, suggesting a transition here from untuned to tuned neurons. Accordingly, **we adopt this criterion: to count as "well-tuned," a multi-unit's mean trial-to-trial correlation must be at least 0.5 standard deviations higher than the mean of the null distribution of correlations.** (The standard deviations are measured in the null distribution.)

We note that this is a more liberal definition for tuning, as can be seen in Fig. R1C: For minimally tuned units, the null and true distributions of trial-to-trial correlations are almost entirely overlapping. (But we note that these distributions are always statistically distinguishable under the Wilcoxon signed-rank test, which is a more liberal criterion still.) Originally, we had required the true distribution's median to be above 90% of the null distribution. We hope that by adopting such a liberal criterion, we will allay Reviewer 1's worry that our results are a consequence of applying an overly strict selection of units.

Adopting this criterion and the new null distribution increases the number of tuned neurons from approximately 250/300 (for speech/monkey vox, resp.) to 400/490. **As expected, including more neurons does lower the median model-neuron correlations mildly (but see next item), but it does so to all models, including STRFs and untrained ANNs, and so does not materially change our results. The exception is the correspondence between deeper layers of the ANNs and non-core neurons, which is now slightly stronger and more visibly obvious; i.e., Fig. 5 is now more compelling.** We attribute this to the inclusion of more non-primary neurons under the new criterion, especially those that are not "core-like." In particular, the new criterion yields 104/174 tuned units, vs. 33/81 under the stricter criterion.

To give the reviewers a feel for the effect of δ, we also reproduce the main figures of the manuscript at $\delta = 1.0$; these are Figs. S5, S6, and S7.

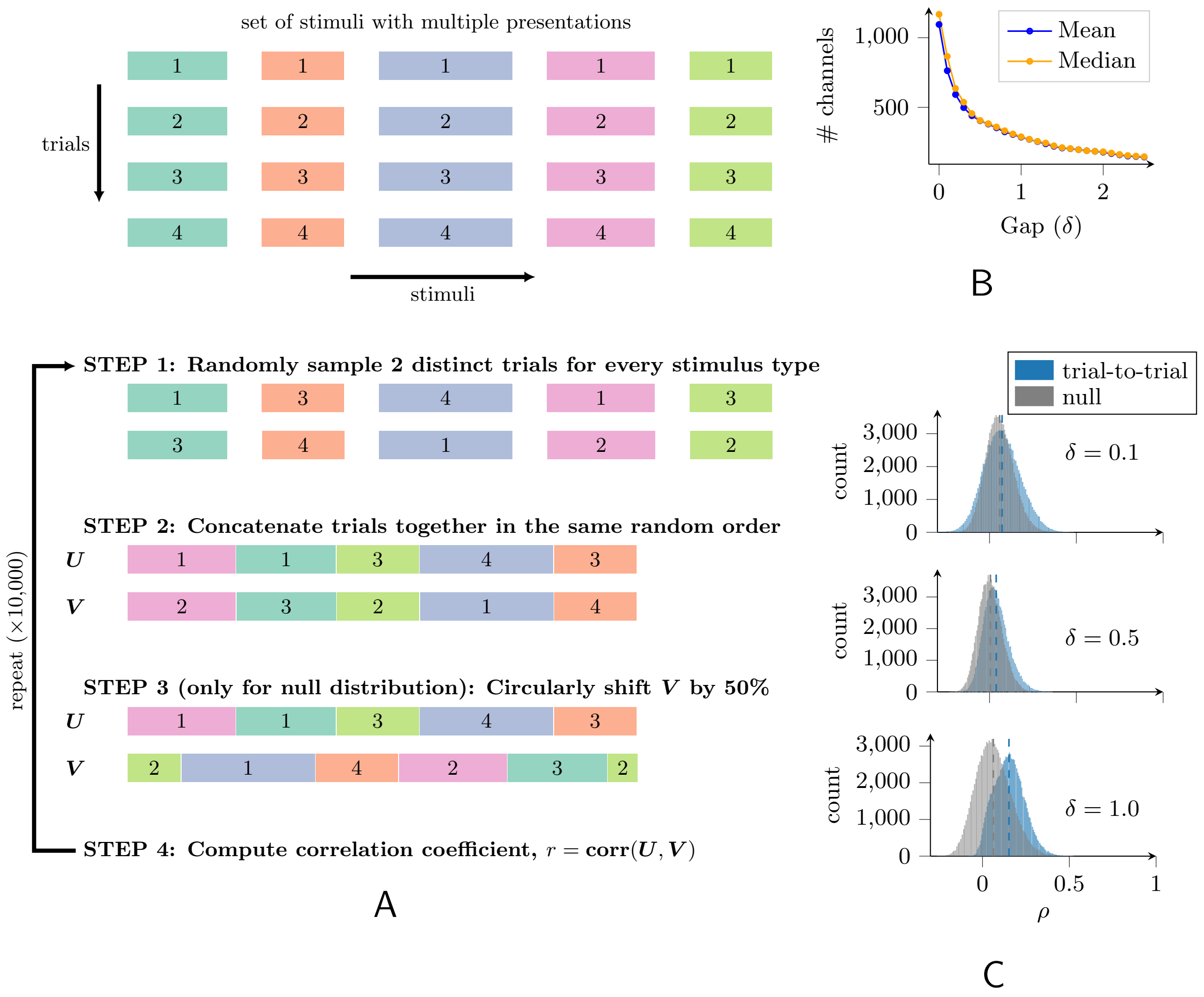

Figure R1: **Noise correction and unit selection.** (A) Procedure for computing the true and null distributions of trial-to-trial correlations. Both distributions are computed using a set of stimuli with multiple presentations. For each stimulus in the set, a distinct pair of trials is randomly selected (STEP 1) and the responses concatenated in random order to form two response sequences, $\boldsymbol{U}$ and $\boldsymbol{V}$ (STEP 2). For computing the null distribution only, $\boldsymbol{V}$ is circularly shifted by half its length (STEP 3), which preserves the marginal response statistics for each channel but destroys any possible temporal correlations with $\boldsymbol{U}$. Finally, the correlation coefficient between $\boldsymbol{U}$ and $\boldsymbol{V}$ is computed (STEP 4). This process is repeated 100,000 times to obtain distributions (true and null) of correlation coefficients. (B) Number of multi-units with at least δ standard deviations between their true and null distributions, as a function of delta. Results are nearly identical if the inter-mean (blue) or inter-median (orange) distance is used. (C) True (blue) and null (gray) distributions of correlations for three randomly selected channels whose means (indicated by dotted vertical lines) are separated by a gap of $\delta = 0.1$, 0.5 and 1.0 times the standard deviation of the null distribution. For all three multi-units, the true distribution is shifted significantly to the right of the null distribution under a Wilcoxon rank-sum test ($p < 0.05$), despite overlapping very heavily (see esp. $\delta = 0.1$).

Improving the TRFs. We fit our temporal receptive fields, for the ANN activities as well as for the amplitude spectrum (i.e., the STRF), starting from the very first time bin in which the audio stimulus is present. Therefore, the first window of features to which the responses are fit contains mostly silence. In the original version of the MS, we accounted for this silence by zero-padding the features. We now instead zero-pad the *audio*, and *then* convert it into features (either by computing the amplitude spectrum or by passing the audio through the ANN), rather than the other way around. This mildly improves the predictive performance of all models, including the STRFs.

Improving the STRFs. We have replaced the STRFs in the original MS with ones based on the cochleagram, since we have found it to outperform the original STRFs in predicting response to both speech and monkey vocalizations (Fig. R2). This does not change the high-level summary. However, previously we had reported that the STRF does not outperform *any* of the trained ANNs at predicting responses to both speech and monkey vocalizations. We must now modify this to say that, for explaining the responses to monkey vocalizations, the STRF does outperform one network (SPEECH2TEXT) and is very close to another (WAV2VEC2).

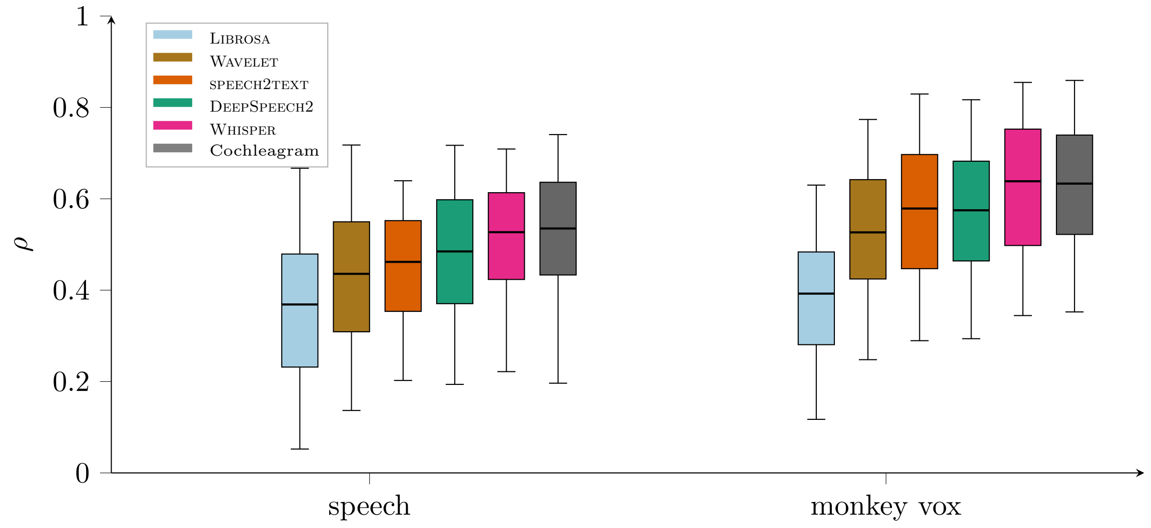

Figure R2: **Comparison of STRF-neuron correlations for various choices of spectrogram computation.** Each box depicts the distribution of correlations across multi-units for a particular choice of spectrogram. The median, middle quartiles, and 5th/95th percentiles are indicated with a black line, shading, and whiskers (resp.). Results are shown separately for each stimulus type, speech and monkey-vocalization (vox).

In addition to these and the changes requested by reviewers, we also rewrote the section of the Discussion on untrained ANNs and STRFs as encoding models, in order to improve its clarity.

Reviewer 1

- *Line 10: "Heretofore, however, the correspondence has only been shown at gross scales, either temporal (∼ 1s) or spatial (1000s of neurons)." There are no references provided. Which works do the authors refer to? Even the original [Yamins et al. 2014] study was performed on a fine time scale, 100ms windows taken 70ms after stimulus presentation, and at a single neuron resolution, which is already a counteexample to this statement.*

  Yes, the array-based papers in visual cortex use ∼100-ms bins. However, the stimuli themselves are static, and there is no notion of time in the DNNs used to predict the corresponding spike count. But we should have made this point more clearly. (We cited no references because we don't think this is allowed in the Author Summary.) We now say, "Heretofore, however, the correspondence has only been shown for **static stimuli**, or at gross scales, either temporal (∼1s) or spatial (1000s of neurons)."

- *Line 78 (and 523): "In particular, we asked how well sequences of spike counts in small (∼50-ms) bins can be (linearly) predicted by different layers of the ANNs." The 50ms bin is gigantic for electrophysiological studies....*

  We take the reviewer's point (although we did put the bin size in parenthesis directly after "small" to eliminate any ambiguity). Since the reviewer refers to 100-ms bins as a "fine time

scale" in his/her first comment, we have adopted that language:

> In particular, we asked how well sequences of spike counts at fine time scales (∼50-ms bins) can be (linearly) predicted by different layers of the ANNs.

- *Line 97: "Electrodes consisted of either 16- or a 64-channel linear probes."—no type / geometry of the probe was provided. It would be useful to understand the limitation of the recording technique (and support claims like line 526 'single "unit" (typically 1–5 neurons)).*

  We now provide the details of probes used as a table in the supplement (S2), and have updated the Methods section accordingly:

  > We used a variety of probes with 1, 16, 32, 48, or 64 channels, selected for each recording session based on which probe was most suitable for accessing the target area on that day. As a result, probe layouts varied across sessions. See Table S2 for details.

- *Line 113: "Spikes were identified by threshold crossing" – what was the threshold? What software was used?*

  We have added the details:

  > Spikes were identified based on threshold crossings, defined as voltage deflections exceeding 3.5 standard deviations from the mean extracellular voltage on each probe. Unthresholded data were collected using Intan software and subsequently thresholded using custom MATLAB code.

- *Lines 120-160: ...It is hard to decipher 1) what correlation was computed (correlation between what and what?).... A figure/equation would be helpful to explain this procedure.*

  We have created such a figure, which we added to the supplement but include here as well for convenience (Fig. R1A).

  We also rewrote the relevant section of the Methods to make all details more explicit:

  > To characterize this variability, we analyzed the response of each multi-unit to the repeated stimuli (see previous). In particular, since sample correlation coefficients converge on the true correlation as the number of samples (in our case, bins) increases, we first concatenated together responses from multiple trials. More precisely (see Fig. R1), for each of the M unique test stimuli, we randomly selected a single pair of responses, (U_m, V_m), with $U_m \neq V_m$, for $m = 1, \ldots, M$. We then concatenated together all M of the U_m into a single "long" sequence, U, and likewise for all M of the V_m into a sequence V, in the same order. Thus for TIMIT, for which the repeat set was 10 unique sentences, each on average 1.6 seconds long, the two concatenated sequences U and V were each about 320 samples long (20 samples/second × 1.6 seconds/sentence × 10 sentences). For the monkey vocalizations, U and V had about 220 samples each (20 samples/second × 1.0 second/vocalization × 11 vocalizations). The sample correlation between U and V provides an estimate of this multi-unit's reliability. To generate more such estimates, we repeated this entire procedure 100,000 times, yielding 100,000 correlation coefficients for each of the ∼1700 multi-units (recording channels).

- *Line 158: "We estimated this correlation with the median of the trial-to-trial response" – why the median? The correction was derived for the mean response.*

  The correction is derived in terms of the mean (expected) *response*, but the distribution from which we pick the median is of *correlation coefficients*. In particular, the correction is derived

in terms of the true (rather than the empirical) correlation, and the theory instructs us to use this single number. (We have added a derivation to the Supplement for completeness.) In practice, of course, we can only measure empirical correlations, which yields a distribution across our resampling procedure; the theory does not give us guidance on how to choose from among these. It might seem that additional theory about the distribution of empirical correlation coefficients could help here, but the distribution we end up with has non-zero variance not just because finite samples are used to estimate the correlation coefficients, but also because the data are unlikely to be exactly identically distributed. We chose the median rather than the mean of this distribution for its robustness against outliers.

Having said that, we re-ran our analyses using the mean rather than the median, and the results are not visibly different. See also Fig. R1. In the new version of the MS, we use means.

- *Line 164: "generating (corrected) correlations much greater than 1.0. This makes all results noisier and harder to interpret." – this alone is a weak justification for applying a stricter selection of units included in the analysis. Perhaps, if the mean correlation was used, the stability would have been better. Was mean > median trial-to-trial correlation for most of the neurons?*

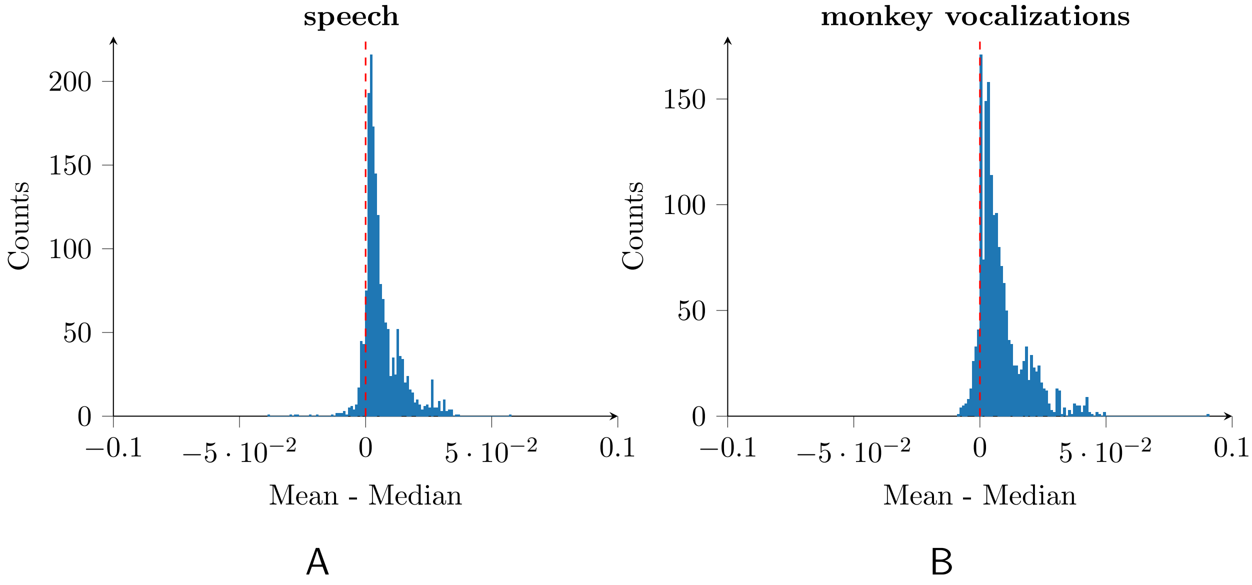

Figure R3: **Distribution of differences of means and medians of trial-trial distributions.**

The mean did tend to be greater than the median, but only by a very small amount. Fig. R3 shows the histogram of differences (mean - median) for trial-to-trial distribution of all recorded units. Using one rather than the other has no visible effect on the results.

In response to the reviewer's concern, we have now loosened the criterion for being "tuned," requiring the median/mean of the true distribution of trial-to-trial correlations to be only 0.5 standard deviations above the median/mean of the null distribution, rather than above 90% of the null distribution. Borderline-tuning is shown in the middle panel ($\delta = 0.5$) of Fig. R1C. In brief, this allows about 60% more units into our analysis, without much changing the high-level results. Please see our **General replies** at the top of this document for a fuller discussion.

- *Line 228: How was resampling (upsampling) done for hidden layers?* We resampled in the Fourier domain using the standard method; to wit, for upsampling, by zero padding

frequencies out to (half) the target frequency and then converting back to the time domain. In practice, we used SciPy's `resample` method to do this.

- *Line 289: The correct term for thresholded activity on a recording channel is a MUA: multy-unit activity. Calling MUA a 'neuron' is misleading.*

  We have replaced the noun "neuron" with "multi-unit" or (where appropriate) "multi-unit activity" throughout.

- *Line 330 (and also repeated in line 372): "Indeed, the most predictive ANN layers correlate with the median neuron at about 0.65 (noise-corrected). This is comparable to the best models of auditory neurons to date [21], which are complex neural networks fit directly to the neural data, whereas our models fit only the linear readout." First, the results from [21] are not presented in this paper, are they? How can we compare [21] to the current paper if they are using a different noise correction (mean rather than median) and different datasets? To justify this claim, one would need to evaluate the model from ref. [21] on the same dataset and apply the same noise correction.*

  Using the mean rather than the median correlation coefficient to normalize doesn't change our results much (see our replies above), but still we agree that the comparison isn't precise, since that study and ours differ in the animal model (ferret in theirs), number of data collected, and other details of the experiments. We have now added a line to the MS saying just this: "Nevertheless, we caution that direct comparisons are difficult, since that study and ours differ in the animal model (ferret in theirs), number of data collected, neuron-inclusion criteria, and other details of the experiments."

- *Figure 1: Impossible to understand what is on the axes, as not a single axis in the plots was labeled. Why are all scatter plots the same? Are these points taken from the data, or drawn randomly for illustration?*

  This is just a schematic (cartoon) plot—not real data. We (re-)used this scatter plot just as an icon for the linear readout. We have added the word "schematic" before "linear plots" in the figure caption, and we have labeled the axes in the embedded schematic plots.

- *Figure 3: It would be useful to add linear decoding from all layers together. It could be that the recorded neural population spans multiple processing layers, so it would be useful to know how single layer prediction compares to all layers together. If all-layer prediction is not so far from the best-layer prediction, then it would also suggest that the layer-by-layer comparison approach, originally developed for mostly feed-forward visual cortex, can be applied to auditory cortex.*

  This is an interesting proposal. We have applied it to Whisper (tiny); see Fig. R4B. It appears that the features provided by the best layer (here, 2) yield almost the exact same encoding performance as the concatenation of features from all layers. (We also verified that the regularization penalty was allowed to be sufficiently large for the all-layers TRF: We allowed this parameter to range between 10^{-5} and 10^{10}, and the optimal penalty found by cross-validation was between 10^{0} and 10^{5} for all the multi-units.)

Reviewer 2

1. *Given the use of six presumably architecturally distinct deep neural networks for prediction, it remains unclear why these specific networks were chosen. A detailed comparison of the performance differences among these networks would provide valuable insight into the modeling outcomes.*

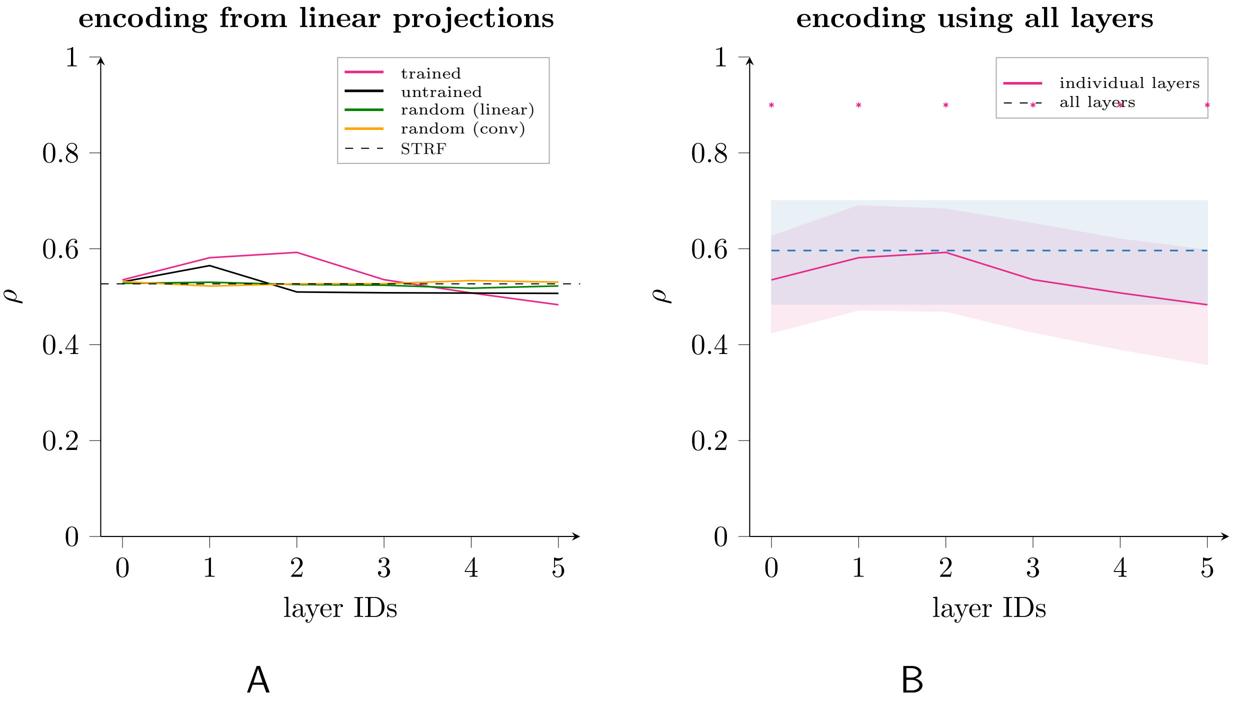

Figure R4: **Encoding from linear projections and multi-layer representations.** (A) Encoding from features obtained by passing spectrogram representations through a stack of linear layers (fully connected layers in green; convolutional layers in yellow). Performance across layers of the trained Whisper (tiny) model is shown in pink, with corresponding re-initialized layers shown in black. Plotted lines indicate the median performance of each distribution. (B) Encoding performance when combining features from all the layers of the Whisper (tiny) model, shown in the blue distribution with the median indicated by a blue dotted line. Performance of individual trained layers is shown in the pink distribution with the median indicated by a solid pink line.

We take the reviewer to be asking about the performances of these ANNs as encoding models (rather than on the ASR task), and in particular connecting differences in performance to the differences in the ANNs. In particular, we wrote in the Methods that our choices of ANN architectures

> ...allowed us to investigate the effects of
>
> - network input: waveform (wav2letter, wav2vec2) vs. spectrogram (the remainder);
> - network architecture: pure convolution (wav2letter), self-attention (wav2vec2, speech2text, Whisper), and recurrent (DeepSpeech2);
> - training: "weak" supervision (Whisper), a combination of supervised and unsupervised learning (wav2vec2), or fully supervised (the remainder);
> - network size (Whisper tiny vs. base)

(This is why we chose these specific ANNs.) But, as the reviewer notes, not all of these played a prominent role in the MS. We have now expanded the Discussion on this point in a new section, "**The effect of the choice of ANN on encoding performance**."

In short, none of the architectural differences seem to be decisive. Instead, the biggest effect on downstream encoding performance seems to be from the size and diversity of the training set, since the consistently best models are DeepSpeech2 and the Whisper models, and these were trained on by far the largest datasets. To investigate this further, we ran our analyses

on a version of WAV2VEC2 that was pre-trained on AudioSet (audio from YouTube videos) rather than LibriSpeech (all English speech) and with no supervised fine-tuning (rather than fine-tuning on LibriSpeech). This does improve the encoding performance of deep layers of WAV2VEC2, but not the overall performance of the model. We refer the reviewer to the Discussion for more on this head.

2. *Beyond neuronal response, comparing additional features such as frequency tuning between neurons and models could offer further understanding.*

   We agree that this is an interesting and useful approach. Indeed, we see it as part of a broader project of carrying out "electrophysiology" on deep neural nets. But we think it deserves full treatment in a MS of its own and would prefer—unless the reviewer thinks it's necessary—not to bury it at the end of this manuscript.

3. *Concerning the hierarchy analysis, it's uncertain whether the hierarchical structure observed in ANN layers mirrors that of the auditory cortex. The current evidence does not strongly support this assertion.*

   With our improved criterion for being tuned (see **General replies**), we end up analyzing more non-core neurons, and the distinction made in Fig. 5 gets stronger. That is, the "preference" for deeper layers of the ANN among non-core neurons is now more pronounced. We also note that the distributions, core and non-core, of "preferred layers" remain statistically significantly different at the other choices for δ that we analyzed ($\delta = 0.3$, 0.5, or 1.0). Thus the finding is robust, even though the effect size is small. We therefore retain our original conclusion that

   > The primary and non-primary distributions are broad compared to the differences between them. This suggests a "soft" anatomical hierarchy, with many neurons in belt and parabelt playing the role of lower-level neurons, and vice versa.

   But we agree that this correspondance is not so stark as in some other studies. We discuss this at greater length in the Discussion section, where we compare it to other results for auditory cortex in the literature (ECoG and fMRI).

4. *Clarification on which subregions of the auditory cortex were recorded from, and specifically which cortical layers, would be beneficial.*

   We have now clarified this in the Methods:

   > Our ideal approach was to advance the probe until the deepest channel no longer registered neural activity. While this generally allowed us to sample across all cortical layers at most recording sites, accessing cortical fields near the edges of the craniotomy often limited our ability to record from all channels on a given probe. Additionally, the electrode insertion angle was not always orthogonal to the auditory cortex, making it difficult to confidently assign specific channels to specific layers. Based on these constraints, we speculate that the majority of the recorded neurons presented here are located within the supragranular and granular layers of the auditory cortex.

5. *More details on the training process of the ANNs, including hyperparameters used, would aid in replicating and understanding the study's results.*

   In the supplements, we now provide extensive details on the ANN performance on ASR tasks (Table S4), training details (Table S5), layer properties (Table S6), and constructions of the spectrogram used as inputs (Table S4). For WAV2LETTER, which we trained "in house," we also provide the training hyperparameters along with links to the code repository and trained

weights. For the other ANNs, which we downloaded pre-trained, we provide links to the relevant repositories (**Pretrained Networks**).

6. *It is unclear if there is a clustering of neurons in Fig. 5. Additional explanation may help address this observation.*

   See our answer to item (3) above.

Reviewer 3

1. *It seems the authors did not apply any multiple testing correction to the results. Given the large number of comparisons made in the study, especially in Fig. 3, it would be important to apply a correction such as controlling the false discovery rate to account for multiple comparisons. This would help to reduce the likelihood of false positives and provide a more accurate assessment of the statistical significance of the results.*

   We originally opted not to adjust the results in Fig. 3 for multiple comparisons because the standard procedures are very conservative when the hypotheses are positively correlated, as they clearly are here for adjacent layers: If layer n predicts neural activity well, then so will layers $n - 1$ and $n + 1$; the curves are fairly smooth. This conservative bias is particularly large when making many comparisons, as in Fig. 3. Put the other way around, the fact that the distributions (e.g., of STRF-neuron and DNN-neuron correlations) are distinguishable at essentially all layers of the DNNs shows that these are not false positives.

   Nevertheless, to be safe, we have now controlled the false-discovery rate with the Benjamini-Hochberg procedure. It is still overly conservative for the reason just given, but in any case has essentially no effect on overall significance: a single comparison (layer 8 of WAV2VEC2 vs. the STRF) loses statistical significance.

   Having said that, we suppose the reviewer might be surprised that these differences are so significant even when (say) the medians of the distributions are visibly very close compared to the interquartile distances of the distributions. Unfortunately, the plot is somewhat misleading in this respect: we are making pairwise comparisons (Wilcoxon signed-rank test), and the distribution of intra-pair differences is much smaller than these interquartile differences, but this cannot be seen in such a plot. In other words, neurons that are explained well by STRFs are typically also explained well, but better, by DNNs; and likewise neurons that are explained poorly by STRFs are explained poorly, but not as poorly, by DNNs. A standard way to exhibit this is with a ladder plot, but that would preclude any compact representation.

   Alternatively, we could plot *ratios* of correlation coefficients. That is, for each multi-unit, we can compute the ratio of the correlation coefficients for the ANN and a baseline—e.g., the STRF. Because this takes pairing into account, the resulting distribution of correlations is more easily seen to be shifted above 1.0 (i.e., the ratio at which the ANN is no better than the STRF). It has the added advantage of canceling out the noisy normalizer, so the correlation distributions are narrower. Its disadvantage is that absolute correlations can not be read off such a plot.

   We think the advantages are sufficiently compelling to include this presentation of the data in a supplementary figure, reproduced here as Fig. R5.

2. *In lines 133-143, the authors describe their approach to construct a null distribution of correlations. However, this distribution assumed Poisson firing and neglects any over- or under-dispersion. Did the authors check the Fano factors of the neurons? If the Fano factors are not close to 1, the null distribution may not be appropriate. It would be helpful to provide more details on whether it is robust to deviations from Poisson firing.*

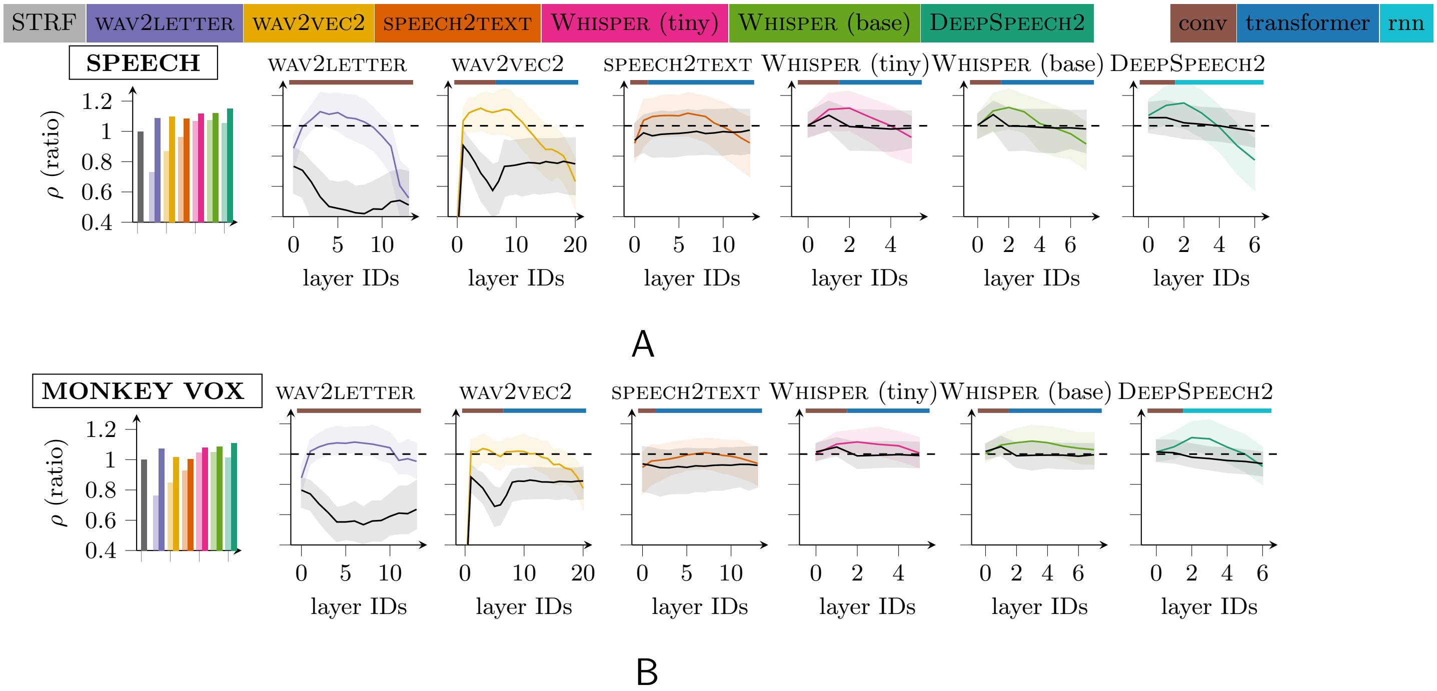

Figure R5: **Model-neuron correlation ratios.** All subpanels show the correlations between model predictions and the multi-unit activity they are supposed to predict, each normalized by the corresponding STRF-neuron correlation. (A) Model-neuron correlation ratios for speech (TIMIT) stimuli. Bar plot: median (across multi-units) correlation ratio for the STRF (gray, necessarily 1.0) and of the best layers of each of the neural networks, both trained (dark colors) and untrained (light colors). Line plots: the distributions of correlation ratios as a function of ANN layer for each of six trained networks (colored) and their untrained counterparts (gray). The median (solid line) and interquartile range (shaded region) are shown. The layer type is indicated along the top of each plot: convolutional (brown), self-attention (blue), and recurrent (light blue). (B) The same as A but using monkey vocalizations for stimuli.

We see the reviewer's point and have accordingly adopted a new strategy for computing the null distribution—see the **General replies** above.

3. *In line 231-232, the authors explain that a 350 ms window of ANN activity was used to predict the neural responses. However, this window is much longer than the typical response times of auditory cortex neurons. It would be interesting to see how the results change with shorter windows, closer to the response times of the neurons.*

   The question of how long the windows should be is not obvious *a priori*. STRFs constructed for primary auditory cortex typically have receptive fields (RFs) of at least 100 ms and frequently as large as 200 ms (see e.g. "A dynamic network model of temporal receptive fields in primary auditory cortex" in this journal, including the explanation therein for how such RFs can be constructed from neurons with short integration windows). For the ANNs, the answer can be quite different: the fraction of the audio "seen by" the 350-ms TRF (linear readout) is greater even than 350 ms, because the ANN units themselves have receptive fields (we have listed them in the supplementary tables).

   Therefore, we treated window length as an empirically determined hyperparameter. Optimizing systematically for it on a per-neuron basis is prohibitively time-consuming. (The fitting procedure, optimized to run on GPUs, already takes about a full day for all networks, layers, neurons, etc.) Informally, we found that the STRF, wav2letter, and DeepSpeech2 improve up to about 250-ms windows. speech2text, Whisper, and wav2vec2 do not improve beyond 100-ms windows, and in fact the Whisper networks get slightly worse. Beyond

250-ms, DeepSpeech2 continues to improve, which is why we originally opted for 350-ms windows.

In the current version of the MS, we now use 250-ms windows, since this is the optimum for STRFs and therefore the most conservative. We could in theory have picked the optimum for each ANN as well, but this would raise questions of fitting to the test data, and is frankly too complicated to justify or explain.

But the overall picture doesn't change much. For comparison, we include here a reprise of Fig. 3 in the main text but with 100-ms windows, Fig. R6.

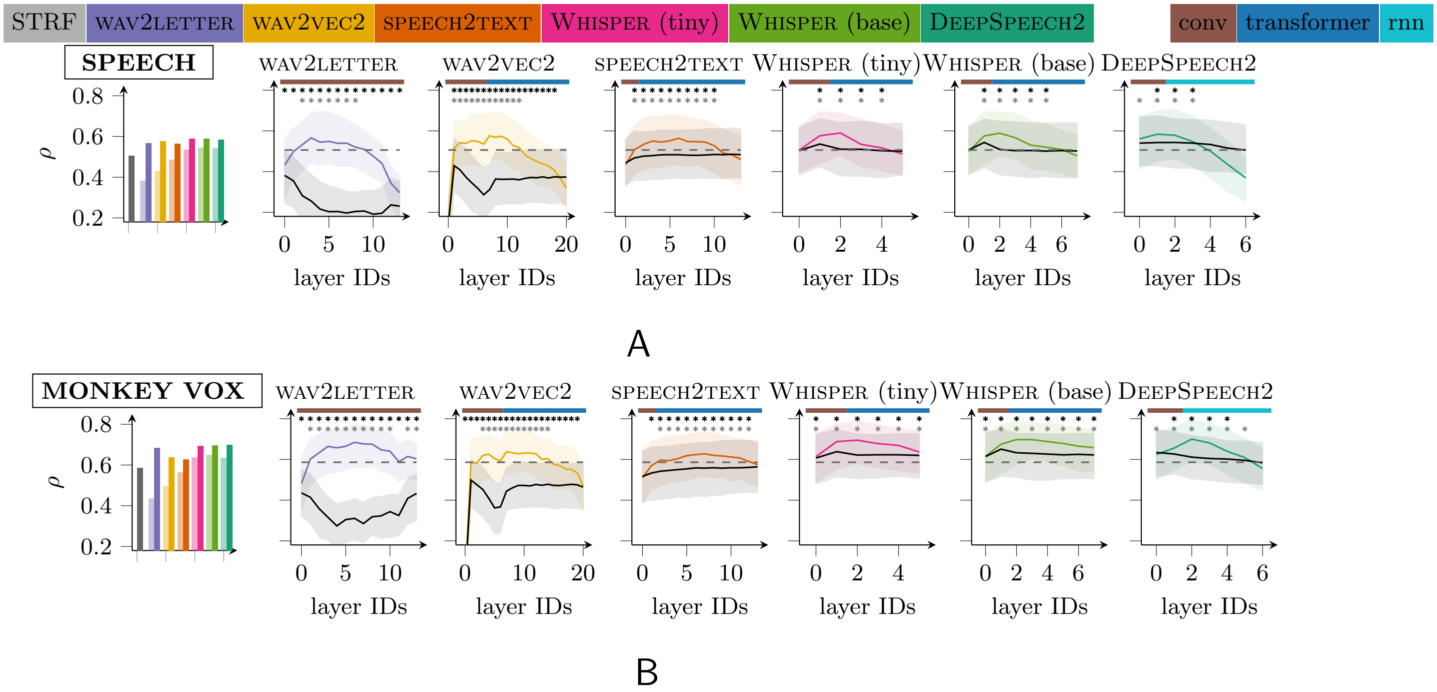

Figure R6: **Model-neuron correlations with 100-ms TRFs.** All subpanels show correlations between model predictions and the multi-unit activity they are supposed to predict. (A) Model-neuron correlations for speech (TIMIT) stimuli. Bar plot: median (across multi-units) correlation of the STRF (gray) and of the best layers of each of the neural networks, both trained (dark colors) and untrained (light colors). Line plots: the distributions of correlations as a function of ANN layer for each of six trained networks (colored) and their untrained counterparts (gray). The median (solid line) and interquartile range (shaded region) are shown. At starred layers, the trained ANN is significantly superior to its untrained counterpart (top row of stars) or a STRF (bottom row of stars; Wilcoxon signed-rank test with $p < 0.01$). The layer type is indicated along the top of each plot: convolutional (brown), self-attention (blue), and recurrent (light blue). (B) The same as A but using monkey vocalizations for stimuli.

4. *In lines 484-491, the authors suggest that the untrained networks are essentially linear transformations of the input. This is an interesting idea that is presented as speculation. However, the idea could be easily tested by comparing to random projections with the same dimensionality. It would be interesting to see if the performance is similar to the untrained networks.*

   To be precise, the nonlinearities are also necessary for model-neuron correlations to *increase* across layers, since stacking linear transformation yields only a single linear transformation. (As we say in the MS, "Furthermore, because the transformations are not *perfectly* linear, some new features can be created....") But as long as the linear transforms do not lose rank, yes, they should not degrade performance. To test this, we added a stack of linear layers—either fully connected and convolutional—and evaluated encoding performance at the output

of each layer. Fig. R4 shows median performance across layers, along with the original (main-text) results for WHISPER (tiny). This confirm that while nonlinearities are necessary to create new features, using only linear projections does not degrade encoding performance.

Minor comments:

1. *Fig. 3: The color code is not immediately clear. A legend would be helpful. Please also label the x-axes. Labeling each row might also help to make the figure easier to interpret.*

2. *Line 382: Fig. 3B → Fig. 3A*

3. *Line 428: the the → the*

4. *S2-S7 Tables: rho columns missing.*

Thank you; we have now corrected these.

Bibliography

[1] Alexander J.E. Kell, Daniel L. K. Yamins, Erica N. Shook, Sam V. Norman-Haignere, and Josh H. McDermott. A Task-Optimized Neural Network Replicates Human Auditory Behavior, Predicts Brain Responses, and Reveals a Cortical Processing Hierarchy. *Neuron*, 98(3):630–644.e16, 2018.
