# Supplementary material for: Deep neural networks explain spiking activity in auditory cortex
Source: PLoS Comput Biol. 2025 Aug 25;21(8):e1013334. doi: 10.1371/journal.pcbi.1013334 (PMC12404638; doi:10.1371/journal.pcbi.1013334)
Supplement: Attachment — Submitted filename: DNNEACreplyToReviewer.pdf [file pcbi.1013334.s020.pdf]

Deep Neural Networks Explain Spiking Activity in Auditory Cortex: Replies to reviewers

The first reviewer had some concerns about the supplementary material, which we address here.

1. *Line 633: Model-neural correlations—this is unusual terminology, which needs to be explained. It does make sense after reading the whole new section, but not at the beginning.*

   We now define the term at the outset:

   > It is therefore customary, when reporting the performance of an encoding model, to normalize the correlations of the model with neural activity ("model-neuron correlations") by the correlations achievable by an *optimal* model."

2. *Line 636: "Intuitively, trial-to-trial correlations involve two noisy variables, whereas model-neuron correlations involve only one (the model is deterministic); and so the latter is just the square root of the former.)" – what does this sentence mean? Correlations always involve 2 noisy variables.*

   Both quantities *vary*, but we wouldn't describe the model as "noisy." Put another way, the variation in the model predictions are due only to variations in the stimulus, whereas variations in the neural response are due to both variations in the stimulus and "noise" in the response. (Of course, the neural "noise" may itself not actually be noise; we noted this in the main text.)

3. *Line 639: "The result is well known but for completeness we prove it here precisely" – are there any references?*

   The same noise ceiling can be found in (e.g.) [ref. 21] in our MS (Pennington and David 2023), under the section "Noise-corrected prediction correlation to evaluate model performance." See also the references therein.

4. *Line 642: "(see figure)" – Figure number and caption missing.*

   We refer to the figure embedded in the preceding paragraph (numbering yet another figure in the supplement felt like overkill). We have moved the figure into the paragraph with the reference and now say, "see the probabilistic graphical model at right."

5. *If the model is deterministic (see p. 2 above), then sigma_u = sigma_x and all derivations make no sense, since correlations would be precisely 1.* We think the reviewer has misunderstood the derivation: U does not correspond to the model. As we say in the text, U and V are "the responses...on two different trials." The correlation between them is the trial-to-trial correlation. The model is X: as we also say in the text, "the optimal model...will recover exactly the noise-free mean response to the stimulus, X."

   (Perhaps the reviewer is surprised that the stimulus doesn't show up in our derivation. In some sense, X also corresponds to the stimulus, or more precisely some deterministic transformation thereof. But the derivation does not require any information about the stimulus itself, only the "noise-free response" to it, X, which by definition the optimal model will find.)
